# Suppression of Osteoclastogenesis by Melatonin: A Melatonin Receptor-Independent Action

**DOI:** 10.3390/ijms18061142

**Published:** 2017-05-26

**Authors:** Hyung Joon Kim, Ha Jin Kim, Moon-Kyoung Bae, Yong-Deok Kim

**Affiliations:** 1 Department of Oral Physiology, BK21 PLUS Project, and Institute of Translational Dental Sciences, School of Dentistry, Pusan National University, Yangsan 626-870, Korea; hjoonkim@pusan.ac.kr (H.J.K.); ya120010@naver.com (H.J.K.); mkbae@pusan.ac.kr (M.-K.B.); 2 Department of Oral and Maxillofacial Surgery, Dental Research Institute and Institute of Translational Dental Sciences, School of Dentistry, Pusan National University, Yangsan 626-870, Korea

**Keywords:** melatonin, osteoclast, RANKL, NF-κB, NFATc1

## Abstract

In vertebrates, melatonin is primarily secreted from the pineal gland but it affects various biological processes including the sleep-wake cycle, vasomotor control, immune system and bone homeostasis. Melatonin has been known to promote osteoblast differentiation and bone maturation, but a direct role of melatonin on osteoclast differentiation is still elusive. The present study investigated the effect of melatonin on the differentiation of macrophages to osteoclasts. The presence of melatonin significantly reduced receptor activator of nuclear factor κB ligand (RANKL)-induced osteoclastogenesis and the siRNA-mediated knockdown of the melatonin receptor failed to overcome the anti-osteoclastogenic effect of melatonin. Although melatonin treatment did not affect the phosphorylation of extracellular signal-regulated kinase (ERK), p38 and c-Jun N-terminal kinase (JNK), it markedly inhibited the activation of NF-κB and subsequent induction of nuclear factor of activated T cell cytoplasmic 1(NFATc1). Thus, our results suggest that melatonin could suppress osteoclast differentiation through downregulation of NF-κB pathway with concomitant decrease in the NFATc1 transcription factor induction. Furthermore, melatonin seems to have an anti-osteoclastogenic effect independent of plasma membrane melatonin receptors. In addition to previously reported properties of melatonin, our study proposes another aspect of melatonin and bone homeostasis.

## 1. Introduction

Melatonin is a ubiquitously present hormone from plants to mammals. The existence of melatonin is reported in almost every living creature including bacteria, macroalgae, plants and vertebrates [1,2,3]. Although melatonin is exclusively secreted from the pineal gland, melatonin affects widespread physiological events in mammals [1,2]. The major role of melatonin is in regulating circadian light-dark cycles of the body, with the peak level of melatonin secreted in the darkness. In addition to its role as a timekeeper, recent experiments have shown the diverse and unexpected functions of melatonin. Melatonin has an anti-apoptotic function and cytoprotective properties against hypoxic circumstance [4,5]. It also participates in memory formation, immune system modulation and blood pressure reduction [6,7]. To sum up, melatonin has neuroprotective, oncostatic, anti-inflammatory and anti-oxidant effects as well as a role in regulating circadian rhythm [8,9,10]. Melatonin exerts its effects by binding to plasma membrane receptors or orphan nuclear receptors. In mammals, there are two different membrane receptors (MT1 and MT2) and one nuclear receptor, retinoic acid receptor related orphan receptor (RZR/RORα) [6,11]. In some cases, melatonin is believed to function through directly binding to intracellular proteins such as calmodulin and tubulin [12]. Generally, the direct binding between melatonin and intracellular target proteins is responsible for the anti-cancer activity of melatonin [6,13]. Therefore, the initiation and/or the subsequent signaling pathways of melatonin is dependent on the cell context.

In mammals, bone is a continually remodeled tissue and the bone homeostasis is precisely regulated by a harmonious activity of bone-resorbing osteoclasts and bone-forming osteoblasts [14,15]. In the bone microenvironment, several types of cells including osteoblasts, stromal cells and lymphocytes provide the two critical osteoclastogenic factors: macrophage colony-stimulating factor (M-CSF) and receptor activator of nuclear factor κB ligand (RANKL) [16,17]. M-CSF guarantees the survival of osteoclast precursor cells, and RANKL essentially drives the osteoclastogenic differentiation of precursor cells [17,18]. The binding of RANKL to its receptor, RANK, triggers the activation of three major mitogen-activated protein kinases (MAPKs) such as c-Jun N-terminal kinase (JNK), extracellular signal-regulated kinase (ERK), and p38 as well as NF-κB-signaling pathways [19,20]. Of note, these multiple signaling cascades provoked by RANKL engagement converge into the activation of “nuclear factor of activated T cell cytoplasmic 1” (NFATc1), the master transcription factor for osteoclastogenesis. The genetic experiments in mice have revealed that the forced expression of NFATc1 alone is sufficient to induce osteoclast differentiation in *c-fos^−/−^* precursor cells even in the absence of RANKL [21,22].

The physiologic role of melatonin of relevance to bone homeostasis has come into the spotlight through recent studies. Melatonin has been shown to promote osteoblasts differentiation via activation of ERK1/2-Runx2 pathways, and these actions of melatonin are MT class melatonin receptor dependent [23,24,25]. In addition, melatonin also works on osteoclasts differentiation and bone-resorbing activity by reducing RANKL expression and increasing osteoprotegerin (OPG) expression in osteoblasts [26,27]. OPG is a decoy receptor for RANKL that acts as a negative regulator of osteoclasts differentiation [25,28]. Considering the coupled activity and communication between osteoclasts and osteoblasts, recent studies have focused on the expression levels of pro-osteoclastogenic cytokine, RANKL and anti-osteoclastogenic cytokine, OPG to give an explanation of melatonin’s inhibitory action on osteoclasts differentiation. In this context, the direct role of melatonin on osteoclasts differentiation was examined in this study.

Here, we report the direct inhibition of osteoclasts differentiation from mouse bone marrow-derived macrophages (BMMs) by melatonin, in which RANKL-induced NF-κB pathways and subsequent NFATc1 expressions were downregulated in the presence of melatonin. Interestingly, it seemed that the inhibitory action of melatonin on osteoclasts differentiation was MT1/MT2 melatonin receptor independent. These data not only demonstrate a previously unapprised role of melatonin but also provide an in-depth understanding of melatonin and bone homeostasis.

## 2. Results

### 2.1. Melatonin Inhibited Receptor Activator of Nuclear Factor κB Ligand (RANKL)-Induced Osteoclastogenesis from Mouse Bone-Marrow Derived Macrophages (BMMs)

**Figure 1:**
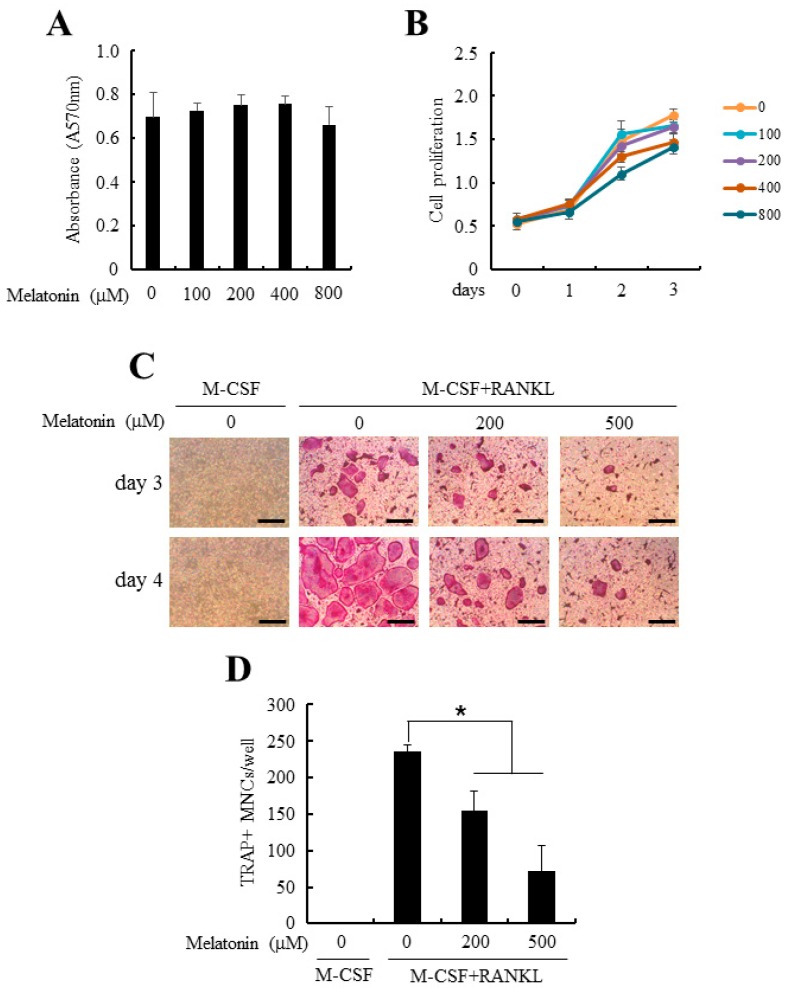
Melatonin suppressed osteoclast differentiation from bone marrow-derived macrophages (BMMs). (**A**) Mouse BMMs were cultured in the presence of indicated doses of melatonin (0–800 µM). After 24 h of culture, cell viability was measured by MTT assay as described in Section 4; (**B**) BMMs were cultured in the osteoclastogenic medium (30 ng/mL macrophage colony-stimulating factor (M-CSF) + 100 ng/mL receptor activator of nuclear factor κB ligand (RANKL)) together with various concentrations of melatonin (0–800 µM) for 3 days. Cell proliferation was evaluated by 3-(4,5-dimethylthiazol)-2,5-diphenyltetrazolium bromide (MTT) assay; (**C**) BMMs were differentiated into osteoclast with osteoclastogenic medium in combination with or without melatonin (0–500 µM) for 4 days. At the end of culture, osteoclasts were stained for tartrate-resistant acid phosphatase (TRAP) activity. Bars, 200 µm; (**D**) TRAP+ multinucleated cells (TRAP+ MNCs) were quantified from experiments in panel (**C**). All quantitative data are presented mean ± standard deviation (SD), * *p* < 0.01).

To examine the direct effects of melatonin on osteoclastogenesis, primary mouse BMMs were purified and allowed to differentiate into osteoclasts with M-CSF and RANKL in the presence of pharmacologic doses of melatonin. Previous in vitro studies with melatonin were performed at 1–500 µM of melatonin [23,26], whereas we looked at the effects of melatonin on cell viability and cell proliferation first. As shown in Figure 1A,B, no cytotoxicity and altered proliferation were observed at examined concentrations (1–800 µM). However, addition of melatonin to the osteoclastogenic culture of BMMs significantly inhibited the formation of tartrate-resistant acid phosphatase (TRAP)+ multinucleated cells (MNCs) in a dose-dependent manner (Figure 1B,C). These data suggest that the anti-osteoclastogenic effect of melatonin was not caused by the toxic effects on precursor cells to differentiate.

### 2.2. Type 1a (MT1) and Type 1b (MT2) Melatonin Receptors are Expressed in Osteoclast Precursor Cells

**Figure 2:**
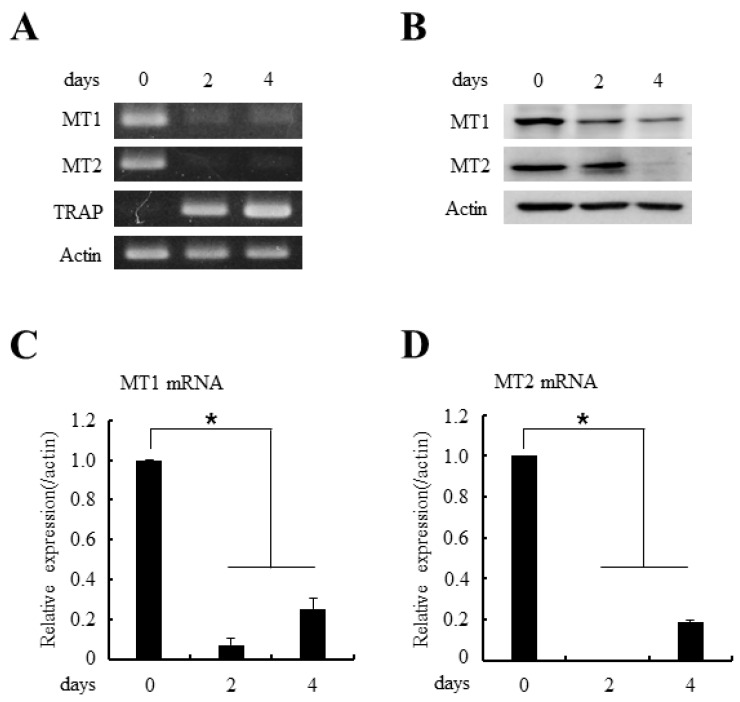
The expressions of MT1 and MT2 melatonin receptors decreased during osteoclast differentiation. (**A**) BMMs were cultured with M-CSF (30 ng/mL) and RANKL (100 ng/mL) for indicated days. After the culture, total RNAs were isolated and the expressions of MT1/MT2 mRNAs were examined by RT-PCR (reverse transcription-polymerase chain reaction) analyses. TRAP served as a marker for osteoclast differentiation; (**B**) BMMs were cultured as in panel (**A**) and whole cell lysates were prepared and MT1/MT2 protein expressions were determined by immunoblotting with anti-MT1 or anti-MT1 antibodies; (**C**,**D**) The mRNA expressions of MT1 and MT2 were analyzed by quantitative real-time PCR after culturing BMMs in the 30 ng/mL M-CSF and 100 ng/mL RANKL for the indicated days. All quantitative data are presented mean ± SD (* *p* < 0.01).

In general, melatonin shows its effects by melatonin receptor activation [11,12,13]. Because melatonin significantly reduced the RANKL-induced osteoclastogenesis, we next examined the expression levels of melatonin receptors during osteoclast differentiation. As expected, the osteoclast precursor cells, BMMs, showed ample expression of both MT1 and MT2 melatonin receptors in mRNA and protein levels (Figure 2A,B, day 0 lane). However, the expressions of MT1/MT2 receptors gradually decreased during osteoclast differentiation (Figure 2A,B, day 2/4 lane). The quantitative real-time Polymerase-Chain Reaction (PCR) analyses further confirmed the decrease in MT1/MT2 mRNAs during osteoclastogenesis.

### 2.3. The Silencing of MT1/MT2 Receptors Failed to Reverse the Anti-Osteoclastogenic Effect of Melatonin

**Figure 3:**
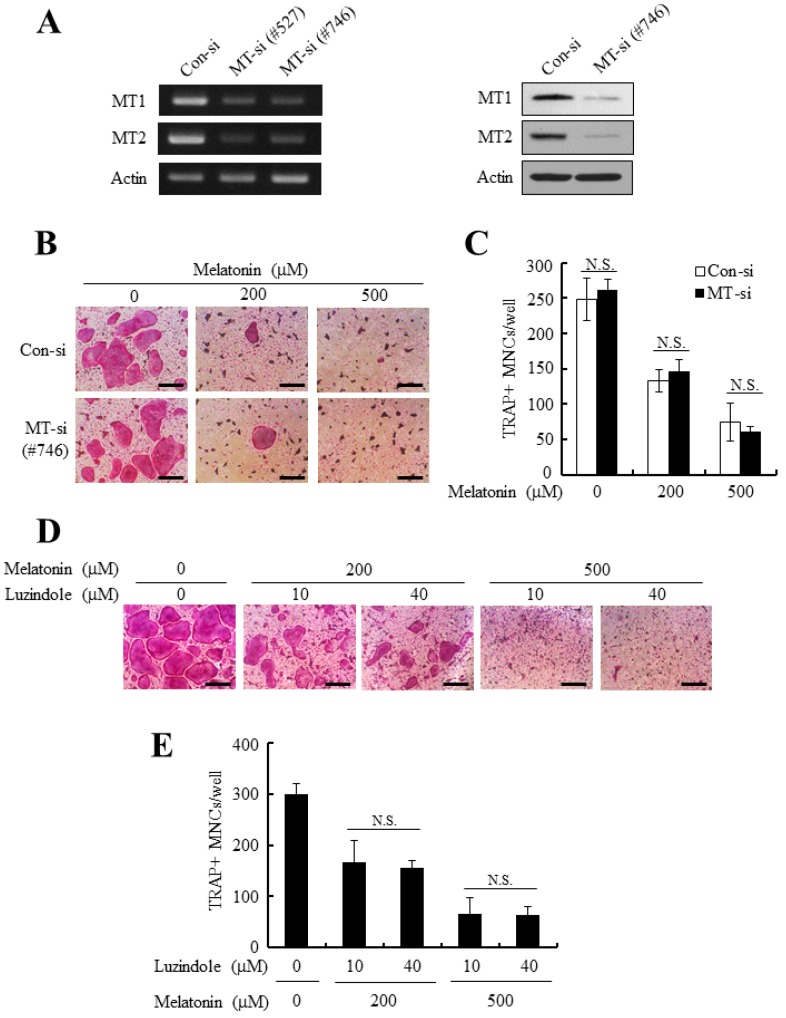
The anti-osteoclastogenic effect of melatonin was not functionally mediated by MT1/MT2 receptors. (**A**) BMMs were transfected with control siRNA (con-si) or MT1/MT2-targeting siRNA (#527, #746) and further cultured for 24 h. The expression of MT1/MT2 was examined by RT-PCR (**left** panel) and Western blotting (**right** panel); (**B**,**C**) MT1/MT2 silenced BMMs were cultured in the osteoclastogenic medium in the presence of melatonin (200 or 500 µM). After a 4-day culture period, cells were stained for TRAP activity and the number of osteoclasts was counted. Bars, 200 µm; (**D**,**E**) BMMs were pretreated with or without luzindole (10 or 40 µM) for 2 h, and osteoclastogenic differentiation was induced with M-CSF + RANKL together with indicated doses of melatonin. At the end of culture, TRAP stained images were photographed and the number of osteoclasts was quantified. Bars, 200 μm. All quantitative data are presented mean ± SD (*p* < 0.01). N.S.: not Significant.

Although the expressions of MT1/MT2 receptors gradually declined during osteoclasts differentiation, at the beginning of osteoclast commitment, the precursor cells (BMMs) had sufficient levels of MT1/MT2, which could mediate anti-osteoclastogenic signals of melatonin (Figure 2). Therefore, the functional contribution of MT1/MT2 receptors to anti-osteoclastogenic effects of melatonin were tested by introducing MT1/MT2-dual targeting siRNAs into BMM osteoclast precursors. The successful and simultaneous knockdown of MT1/MT2 receptors was achieved by #527 and #746 siRNA sequences (Figure 3A). However, the observed reduction of osteoclasts formation after melatonin addition was consistently similar to that of con-siRNA transfected cells (Figure 3B,C). Luzindole has been suggested as a competitive melatonin membrane receptor antagonist [6,29]. To further examine the role of MT1/MT2 receptors for anti-osteoclastogenic effect of melatonin, BMMs were pretreated with luzindole (10 or 40 µM) and allowed to differentiate into osteoclasts in the presence of melatonin. In accordance with MT1/2-knockdown experiments, luzindole treatment did not significantly reverse the anti-osteoclastogenic effect of melatonin (Figure 3D,E). These data suggest that the melatonin inhibits osteoclasts differentiation independent of MT1/MT2 membrane receptors.

### 2.4. Melatonin Had Little Effect on the RANKL-Induced Activation of Three Major Mitogen-Activated Protein Kinases (MAPKs) but Markedly Blocked Nuclear Factor of Activated T Cell Cytoplasmic 1 (NFATc1) Induction

**Figure 4:**
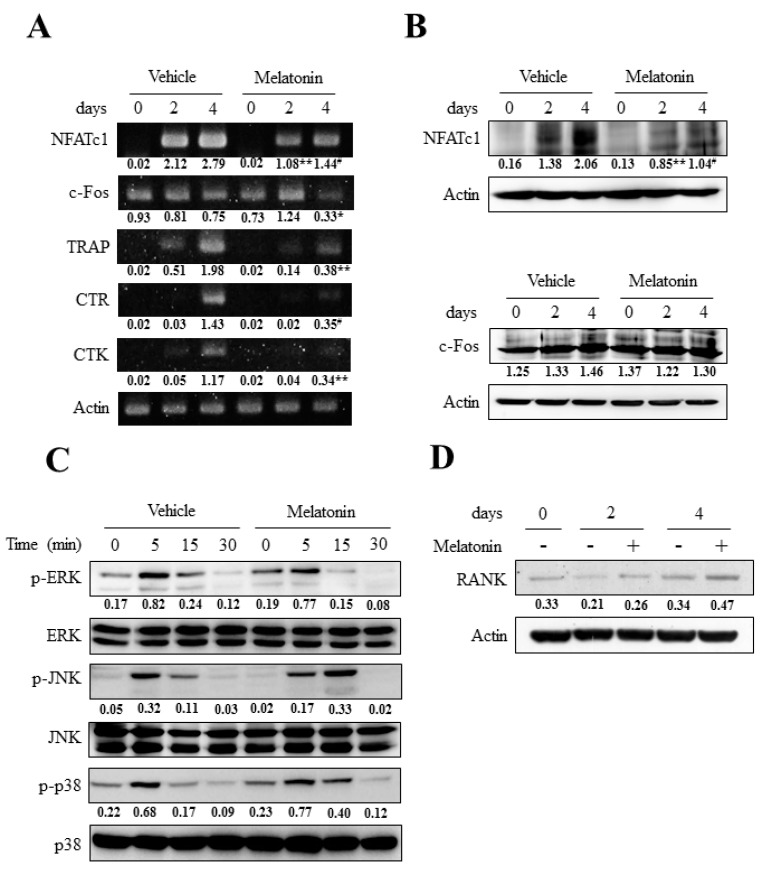
Melatonin showed little effect on RANKL-induced MAPKs activation but significantly reduced NFATc1 expression. (**A**) BMMs were cultured with osteoclastogenic medium (30 ng/mL M-CSF + 100 ng/mL RANKL) for the indicated days in the absence or presence of melatonin (500 µM). The mRNA expressions of NFATc1, c-Fos, TRAP, CTR (calcitonin receptor), and CTK (cathepsin K) were determined by RT-PCR analyses. TRAP, CTR, and CTK were served as a osteoclastic differentiation marker; (**B**) BMMs were cultured as in panel (**A**) and the NFATc1 expression (**upper** panel) and c-Fos expression (**lower** panel) were examined by immunoblotting; (**C**) BMMs were serum starved for 5 h, pretreated with vehicle or melatonin (500 µM) for 2 h, and stimulated with RANKL (100 ng/mL) for the indicated times. Cell lysates were subjected to Western blotting using phospho-specific antibodies against ERK, JNK, and p38. The equal loadings of samples were probed by ERK, JNK, and p38 antibodies, respectively; (**D**) BMMs were cultured as in panel (**A**) and the protein expression of RANK was determined by immunoblotting. Numbers represent the relative band intensity of indicated bands normalized to that of control (actin or respective un-phosphorylated MAPKs) by densitometry. * *p* < 0.05; ** *p* < 0.01; ^#^ *p* < 0.001 versus same time sample of vehicle.

To define the molecular mechanisms underlying melatonin’s anti-osteoclastogenic effect, we next sought to investigate the pivotal signaling pathways for osteoclast differentiation. Among the transcription factors required for osteoclast differentiation, c-Fos and NFATc1 were known to be critical for osteoclastogenesis [16,17,18,19]. Although the mRNA expression of c-Fos was significantly decreased by melatonin (Figure 4A), the protein level of c-Fos was unchanged upon melatonin treatment (Figure 4B lower panel). Importantly, melatonin significantly reduced the induction of NFATc1 from day 2 to day 4 after RANKL stimulation (Figure 4A,B upper panel). In concert with the reduction in NFATc1 level, the expression of osteoclast marker genes such as TRAP, calcitonin receptor (CTR) and cathepsin K (CTK) were significantly decreased compared to when BMMs were treated with melatonin (Figure 4A). The activation of three major MAPKs (ERK, JNK and p38) is an important signaling mechanism involved in osteoclast differentiation. More importantly, ERK has been found to be critical to NFATc1 induction through ERK-AP1 pathways [19,30]. Thus, we next focused on the effects of melatonin upon the activation of ERK and the other two MAPKs to further dissect the upstream pathways which lessened the NFATc1 expression upon melatonin treatment. Contrary to our expectation, there were no conspicuous differences in MAPKs activation between vehicle and melatonin treated cells (Figure 4C). Although it is not statistically significant, the MAPKs pathways showed rather increasing tendency in the presence of melatonin (Figure 4C). However, the treatment of MAPKs inhibitors (U0126 for ERK, SP600125 for JNK, and SB203580 for p38) did not reverse the melatonin-mediated suppression of osteoclast differentiation (data not shown). In addition, melatonin treatment did not significantly affect the expression of RANK (Figure 4D). Therefore, it is likely that melatonin suppressed osteoclast differentiation by inhibiting NFATc1 induction but the reduction of NFATc1 did not result from altered MAPKs activation or reduced RANK expression.

### 2.5. Melatonin Inhibits NF-κB Activity in RANKL-Stimulated Bone Marrow-Derived Macrophages (BMMs)

**Figure 5:**
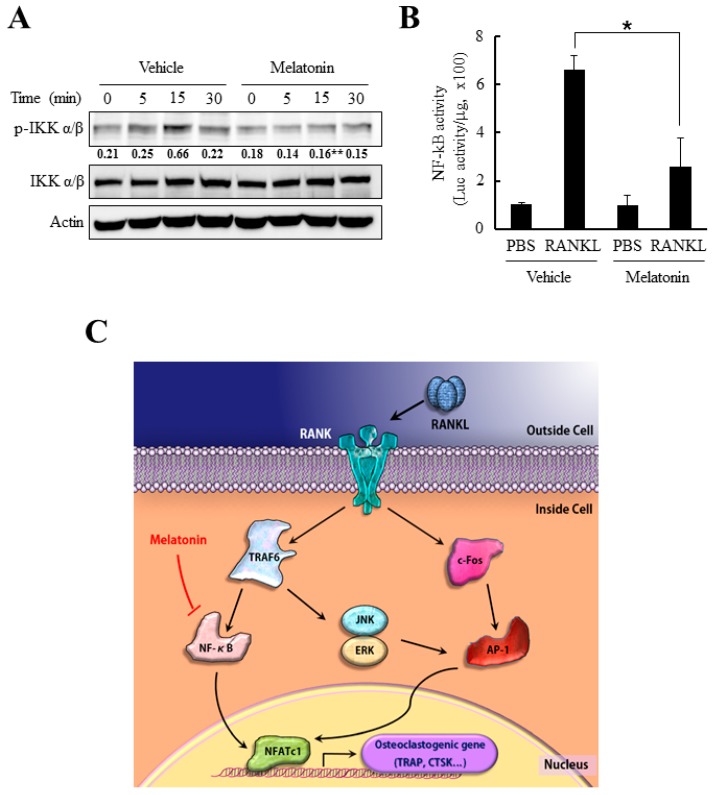
Melatonin reduced the RANKL-induced NF-κB activation. (**A**) Serum-starved BMMs were pretreated with melatonin (500 µM) for 2 h and stimulated by RANKL (100 ng/mL) for 30 min. Cells were lysed and the activation of NF-κB pathway was examined by immunoblotting with an antibody for p-IKK (Phospho-Inhibitory κ B kinase) α/β or IKK α/β. Numbers represent the relative band intensity of indicated bands normalized to that of actin by densitometry (** *p* < 0.01 versus same time sample of vehicle); (**B**) BMMs were transfected with NF-κB dependent luciferase reporter construct. At 24 h after transfection, transfected cells were serum starved for 5 h and stimulated by 100 ng/mL of RANKL for another 24 h. Cells were lysed and relative NF-κB activity was presented by luciferase activity per microgram of protein ( * *p* < 0.05 versus vehicle); (**C**) Schematic diagram for suppression of osteoclast differentiation by melatonin. The ligation of RANK by RANKL triggered activation of downstream signals including NF-κB, JNK, ERK, and c-Fos which converged into the induction of master transcription factor for osteoclastogenesis, NFATc1. Melatonin impeded the activation of NF-κB and subsequent induction of NFATc1 which lead to suppression of osteoclast differentiation.

Since NFATc1 mRNA and protein expression levels were substantially reduced after melatonin treatment without significant changes in the MAPKs pathways, we next examined another important component in NFATc1 induction pathway, NF-κB. NF-κB activation not only positively regulates the expression of many genes involved in osteoclastogenesis (such as TRAP, MMP9, and cathepsin K), but also plays a role in NFATc1 induction in response to RANKL [20,31]. Generally, RANKL-induced NF-κB activation initiates from phosphorylation of IκB (inhibitory κ B), which leads to poly-ubiquitination mediated degradation of IκB [20,31]. To test the effect of melatonin on NF-κB pathway, BMMs were stimulated with RANKL after pretreatment of melatonin. As shown in Figure 5A, the phosphorylation of IκB was significantly reduced by melatonin. Notably, the luciferase reporter assay for the NF-κB-dependent promoter region consistently revealed a substantial decrease in NF-κB promoter activity in the presence of melatonin (Figure 5B). Therefore, it is reasonable to assume that melatonin attenuated NFATc1 induction by inhibiting the RANKL-mediated NF-κB activation, resulting in a suppression of osteoclastogenesis.

## 3. Discussion

Since the discovery of melatonin (*N*-acetyl-5-methoxytryptamine) in the extracts of bovine pineal gland, its physiologically versatile effects have become a matter of particular interest. More importantly, the findings that many tissues in our body other than pineal gland could produce melatonin have added emphasis on the physiological functions of melatonin. Melatonin plays a role in multiple physiologic systems in our body. Melatonin affects the sleep-wake system, psychiatric system, endocrine system, immune modulatory system, and cardiovascular system [1,2,3,4]. In addition, melatonin also has effects on pathologic conditions such as obesity, diabetes, Alzheimer’s disease, and cancer [6,7,8,9]. Although the beneficial pleiotropic actions of melatonin have been reported, melatonin is less well known, considering its widespread use to counter jet lag and insomnia. A direct osteogenic effect of melatonin has been demonstrated by in vitro and in vivo studies. In the presence of melatonin, the osteoblast differentiation was enhanced in pre-osteoblast cells and osteosarcoma cell lines with increasing expressions of osteoblast marker genes including alkaline phosphatase, osteopontin, bone sialoprotein, and osteocalcin [3,25]. Because the activities of osteoblasts and osteoclasts are coupled with one another, melatonin also brings about the inhibition of bone resorption by downregulating the synthesis and release of RANKL from osteoblasts [26]. Collectively, melatonin seems to cause augmentation of bone quality through the regulation of both osteoblasts and osteoclasts activity. This bone enhancing effect of melatonin is physiologically plausible since the plasma level of melatonin decreases with age, especially in post-menopausal women. Thus, the decreased melatonin level could be involved in the onset of osteoporosis in the elderly [3,25,26,27]. Although the direct effects of melatonin on osteoclast differentiation were reported by previous studies, these studies were performed with macrophage cell line RAW 264.7 cell [32] or teleost scale osteoclast [33]. The direct effects of melatonin on the osteoclastogenesis of primary mammalian cells remains to be elucidated. In the present study, we showed that melatonin inhibited RANKL-dependent osteoclast differentiation from mouse bone marrow-derived macrophages.

The previous in vitro studies showing bone anabolic or catabolic effects of melatonin were performed at 1–500 µM of melatonin [26,32]. Actually, this in vitro effective concentration of melatonin is higher than the physiological serum levels of melatonin, which is between 0.4 and 0.9 nM [34]. However, it has been reported that oral administration of melatonin (80–240 mg/day) raises the serum concentrations of melatonin 350 to 10,000-fold to micro-molar levels [35] and the bone marrow concentrations of melatonin is maintained 100 times higher than that of serum [36]. More importantly, we first screened increasing doses of melatonin to determine their effect on osteoclast differentiation and we have found that high doses of melatonin did not appear to cause toxic side effects in BMMs. The maximal 800 µM of melatonin showed no cytotoxicity and did not alter the proliferating activity of BMMs (As shown in Figure 1A,B). Relatively lower concentrations of melatonin (1–10 µM) failed to inhibit osteoclastogenesis but 50 µM of melatonin starts to show slight anti-osteoclastogenic activity and above 200 µM of melatonin showed confirmatory anti-osteoclastogenic activity. Therefore we have used 200 or 500 µM of melatonin in this study.

Major actions of melatonin are mediated by specific plasma membrane receptors MT1 and MT2. They belong to the GPCR (G-protein coupled receptor) family typically containing seven membrane-spanning domain. For example, the roles of melatonin on several immunological responses, circadian rhythm regulation, and vasomotor control are mediated by MT1 and MT2 [1,2,3]. Melatonin also exerts its effects by binding to the RZR/ROR class of orphan nuclear receptors. The immunomodulatory effects, circadian effects, and anti-cancer effects of melatonin are known to be partly mediated by these nuclear melatonin receptors [12,13]. Even melatonin could function as a receptor-independent free radical scavenger, which has been addressed by numerous investigations about the radical detoxification and anti-oxidative protection of melatonin [3,9]. Melatonin’s multiple modes of action have made it difficult to understand the signal transduction mechanisms of melatonin underlying various biological activities. In the present report, the anti-osteoclastogenic effect of melatonin was not reversed by MT1/MT2 receptor silencing or melatonin receptor antagonist treatment (Figure 3). Therefore, it seemed that the inhibitory action of melatonin on osteoclastogenesis was irrelevant to MT1/MT2 membrane receptors. Even if the inhibitory action of melatonin was not mediated at least by MT1/MT2 receptors, the precise molecular target of melatonin in osteoclast and underlying mechanisms needs to be investigated by further studies.

Recent reports suggest the critical role of NFATc1 in osteoclast differentiation. NFATc1 is the master transcription factor for osteoclastogenesis that was definitely proved by the experiment, which showed the functional osteoclast differentiation even in the absence of RANKL in NFATc1 overexpressed cells [22,37]. The expression of NFATc1 in RANKL-stimulated BMMs is potentiated by initial NF-κB pathway and ERK-c-Fos pathway [14,31]. Here, we report the significant reduction in the transcriptional activity of NF-κB by melatonin, which comprises the important pathway for NFATc1 induction (Figure 5B). Our results suggest that the inhibition of osteoclast differentiation by melatonin might target the NF-κB pathway that is upstream of NFATc1.

To summarize, the present study showed the previously unappreciated role of melatonin in bone homeostasis. Melatonin directly suppressed osteoclast differentiation through downregulation of NF-κB pathway and subsequent NFATc1 transcription factor induction. Notably, the anti-osteoclastogenic effect of melatonin was believed to independent of plasma membrane melatonin receptors MT1/MT2. Since melatonin also has been known to increase osteoblastogenesis and bone maturation, it might be beneficial for the use of melatonin in bone-resorption associated diseases. Our data not only suggest a novel physiologic role of melatonin, but also provide the pharmacologic efficiency of melatonin administration for preventing pathological bone resorption.

## 4. Materials and Methods

### 4.1. Reagents

Melatonin (*N*-acetyl-5-methoxytryptamine) and luzindole were purchased from Sigma-Aldrich (St. Louis, MO, USA) and dissolved in dimethyl sulfoxide (DMSO). Recombinant RANKL and M-CSF were purchased from PeproTech (Rocky Hill, NJ, USA). Antibodies against phospho-ERK, ERK, phosphor-JNK, JNK, phosphor-p38, p38, phosphor-IκBα/β, and IκBα/β were obtained from Cell Signaling Technology (Danvers, MA, USA). Anti-NFATc1, anti-c-Fos, anti-RANK, anti-MT1 (MEL-1A-R; V-15), and anti-MT2 (MEL-1B-R; T-18) antibodies were purchased from Santa Cruz Biotechnology, Inc. (Santa Cruz, CA, USA). The Leukocyte Acid Phosphatase (TRAP) kit and anti-β-actin antibody were obtained from Sigma-Aldrich.

### 4.2. Isolation of Macrophages from Mouse Bone Marrow and In Vitro Osteoclast Differentiation

Bone marrow-derived macrophages (BMMs) were isolated as described previously [30,38]. Briefly, mouse whole bone marrow cells were collected from femora and tibiae of 5-week-old ICR mice and cultured overnight on 100-mm dishes in α-modified Eagle Medium (α-MEM; WelGENE Inc., Daegu, Korea) supplemented with 10% fetal bovine serum (FBS). After discarding adherent stromal cells, the floating cells were further cultured in the presence of M-CSF (30 ng/mL) on Petri dishes. Cells became adherent after a 3~4 day culture period and were used as BMMs, the osteoclast precursor cells. Animal experiments were approved by the Committees on the Care and Use of Animals in Research at Pusan National University. To induce osteoclast differentiation, BMMs (4 × 10^4^ cells/48-well plates) were cultured with osteoclastogenic medium (30 ng/mL M-CSF + 100 ng/mL RANKL) for 4 days. After the culture, cells were stained for TRAP activity and the TRAP-positive multinucleated cells with ≥3 nuclei were counted as osteoclast. 

### 4.3. Cytotoxicity Measurement and Cell Proliferation Assay

The effects of melatonin on both cell viability and proliferation were examined by a well-established colorimetric assay using 3-(4,5-dimethylthiazol)-2,5-diphenyltetrazolium bromide (MTT, Sigma-Aldrich). In brief, BMMs were seeded in 96-well plates with indicated concentrations of melatonin for up to 3 days. At the end of culture, cells were further incubated with MTT solution (medium containing 0.5 mg/mL MTT) for more 4 h. After the culture, the formation of blue formazan product was measured with microplate reader at a wavelength of 570 nm.

### 4.4. Gene Knockdown by siRNA Transfection

The pre-designed siRNA duplexes for MT1, MT2, and the universal negative control were purchased from GenePharma (Shanghai, China). Oligonucleotide siRNA duplexes were transfected into BMMs with jetPRIME siRNA transfection reagent (Polyplus, New York, NY, USA) according to the manufacturer’s instructions. The efficiency of transfection was >80% when measured by FAM-labeled siRNA control transfection.

### 4.5. Reverse Transcriptase Polymerase-Chain Reaction (RT-PCR) and Real-Time PCR Analyses

For RT-PCR analysis, total RNAs were isolated using TRIzol Reagent (Invitrogen, Waltham, MA, USA) and 1.5 µg of RNAs were reverse-transcribed with Superscript II (Invitrogen) according to manufacturer’s protocol. For real-time PCR analysis, 2 µg of cDNAs were amplified with SYBR green PCR master mix (Applied Biosystems, Forster City, CA, USA) in a MicroAmp optical tube (Applied Biosystems) for 40 cycles of denaturation (15 s) at 95 °C and amplification (60 s) at 60 °C in AB7500 instruments (Applied Biosystems). The primer sets used in PCR were as follows: MT1, 5′-CCATTTCATCGTGCCTATG-3′ (forward) and 5′-GTAACTAGCCACGAACAGC-3′ (reverse); MT2, 5′-GAGTGATTTGCGCAGTTTCC-3′ (forward) and 5′-GAGAGCACCTTCCTTGACAG-3′ (reverse); TRAP, 5′-ACTTCCCCAGCCCTTACTACCG-3′ (forward) and 5′-TCAGCACATAGCCCACACCG-3′ (reverse); NFATc1, 5′-TGCTCCTCCTCCTGCTGCTC-3′ (forward) and 5′-CGTCTTCCACCTCCACGTCG-3′ (reverse); c-Fos, 5′-ATGGGCTCTCCTGTCAACAC-3′ (forward) and 5′-GGCTGCCAAAATAAACTCCA-3′ (reverse); CTR, 5′-TGCATTCCCGGGATACACAG-3′ (forward) and 5′-AGGAACGCAGACTTCACTGG-3′ (reverse); CTK, 5′-AGGCGGCTATATGACCACTG-3′ (forward) and 5′-CCGAGCCAAGAGAGCATATC-3′ (reverse); Actin, 5′-CGATGCCCTGAGGCTCTTTT-3′ (forward) and 5′-GGGCCGGACTCATCGTACTC-3′ (reverse).

### 4.6. Western Blotting

Western blotting was conducted following a standard procedure. Briefly, BMMs were disrupted in a RIPA lysis buffer (50 mM Tris; pH 8.0, 150 mM NaCl, 0.5% sodium deoxycholate, 1.5 mM MgCl_2_, 1 mM EGTA, 1% Triton X-100, 10 mM NaF and complete protease inhibitor cocktail). The protein concentrations of cell lysates were determined using a detergent-compatible protein assay kit (Bio-Rad Laboratories, Hercules, CA, USA) and 30–45 µg of cell lysates were resolved by 8–10% sodium dodecyl sulfat (SDS)-polyacrylamide gel electrophoresis. Separated proteins were transferred onto nitrocellulose membranes and membranes were blocked with 5% skim milk for 1 h. After incubation with appropriate primary/secondary antibodies, the immunoreactivity of membranes was detected with chemiluminescence reagents. The band results were obtained from three independent experiments and quantified using ImageJ software (version 1.50i, National Institutes of Health, Bethesda, MD, USA) by comparing band intensities (relative band intensities normalized to actin or respective control blot).

### 4.7. NF-κB Luciferase Reporter Assay

BMMs were seeded at 5 × 10^5^ cells/well in 6-well plates and transfected with 4 µg of NF-κB-dependent luciferase reporter vector using Lipofectamine 2000 (Invitrogen). After 24 h incubation, transfected cells were detached and re-plated in 96-well plates at 2 × 10^4^ cells/well. Cells were serum starved for 5 h and stimulated by 100 ng/mL of RANKL for 24 h. Cells were lysed in Reporter Lysis Buffer (Promega, Madison, WI, USA), and luciferase activity was examined using a luminometer. Data are presented as luciferase activity per microgram of protein.

### 4.8. Statistics

Data were obtained from at least three independent experiments. The Student’s *t*-test was used to determine the significance of differences between two groups. Differences with *p* < 0.01 were regarded as significant and denoted as an asterisk. Comparative analysis of the groups was performed by ANOVA followed by Student Knewman-Keuls post hoc tests for results in Figure 4 and Figure 5 (* *p* < 0.05; ** *p* < 0.01; ^#^ *p* < 0.001).

## Abbreviations

RANKL	Receptor activator of nuclear factor κB ligand
M-CSF	Macrophage-colony stimulating factor
BMM	Bone marrow-derived macrophage
TRAP	Tartrate-resistant acid phosphatase
